# TRPV6-related intrauterine calciopenic rickets: a case report and literature review

**DOI:** 10.1530/EDM-25-0192

**Published:** 2026-04-08

**Authors:** Geoffrey Chek-Fei Yu, Queenie Wing-Shan See, Mimi Tin-Yan Seto

**Affiliations:** ^1^ Department of Paediatrics and Adolescent Medicine, Queen Mary Hospital, Hong Kong; ^2^ Department of Paediatrics and Adolescent Medicine, The University of Hong Kong, Hong Kong; ^3^ Department of Obstetrics & Gynaecology, Queen Mary Hospital, Hong Kong

**Keywords:** TRPV6 variant, neonatal hyperparathyroidism, skeletal dysplasia, vitamin D supplementation

## Abstract

**Summary:**

We report a female Chinese neonate with antenatal rickets and secondary hyperparathyroidism who was found to have novel compound heterozygous *TRPV6* variants (c.1160G>A and c.658C>T). She was treated with calcium, phosphate and vitamin D supplementation, achieving full biochemical and radiographic recovery by age two years. The review of all 14 reported cases confirms a consistent phenotype of neonatal secondary hyperparathyroidism with universal parathyroid hormone (PTH) elevation, frequent fractures and respiratory distress. Excellent outcomes are achieved with supplementation. The correlation between vitamin D deficiency and hypocalcaemia in these neonates suggests that maternal vitamin D status may modify disease severity.

**Learning points:**

- *TRPV6* mutations should be considered a rare cause of neonatal prenatal rickets and secondary hyperparathyroidism, even in the presence of normocalcaemic hyperparathyroidism.
- Prognosis is excellent with early mineral and vitamin D supplementation.
- Multidisciplinary care is essential for diagnosis, management and follow-up.
- Maternal vitamin D status may modulate disease severity, suggesting that prenatal supplementation could be beneficial.

## Background

TRPV6-related intrauterine calciopenic rickets is exceedingly rare, with only 12 cases reported worldwide prior to this report. TRPV6, a member of the TRP channel family, is distinguished by its high calcium selectivity. This channel is implicated in energy-dependent calcium transportation processes, including intestinal absorption, sperm maturation and placental transfer. A defective TRPV6 channel will cause severe hypocalcaemia and metabolic bone disease in the newborn. Herein, we report a newborn girl with *TRPV6* variants who presented with hyperparathyroidism and antenatal rickets.

## Case presentation

**Figure 1:**
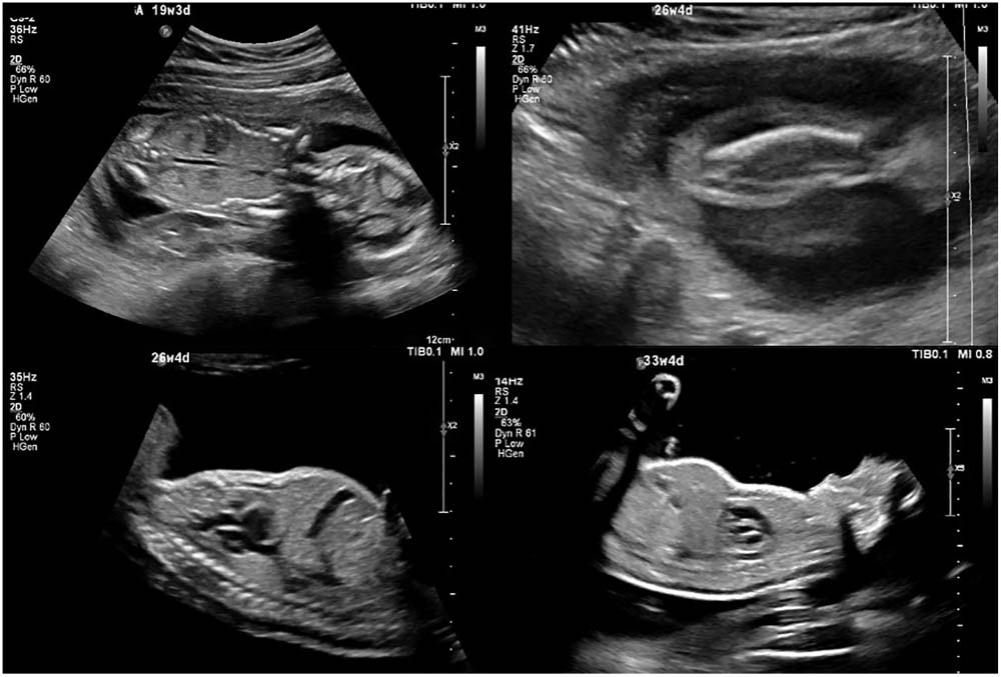
Bell-shaped thorax at 19 + 3, 26 + 4 and 33+4 weeks gestation. Short ribs with wavy deformity at 26+4 weeks gestation. Bowed fibula without obvious fracture.

A female neonate was born to a non-consanguineous Chinese family at 37 weeks and 2 days of gestation via normal spontaneous vaginal delivery. Antenatal ultrasound identified short long bones, a bell-shaped thoracic cage and polyhydramnios (Fig. 1), prompting a referral for genetic investigation. Antenatal trio whole-exome sequencing revealed compound heterozygous variants of uncertain significance (VUS) in the TRPV6 gene: a maternally inherited c.1160G>A (p.Cys387Tyr) and a paternally inherited c.658C>T (p.Arg220Trp).

The infant required transient respiratory support with continuous positive airway pressure (CPAP) and positive pressure ventilation (PPV) immediately after birth for shallow breathing and carbon dioxide retention. Apgar scores were 8 and 10 at 1 and 5 min, respectively. She was small for gestational age (SGA) with a birth weight of 2.225 kg (3rd percentile), length of 42 cm (>6 cm below the 3rd percentile) and head circumference of 31 cm (3rd percentile).

## Investigations

**Figure 2:**
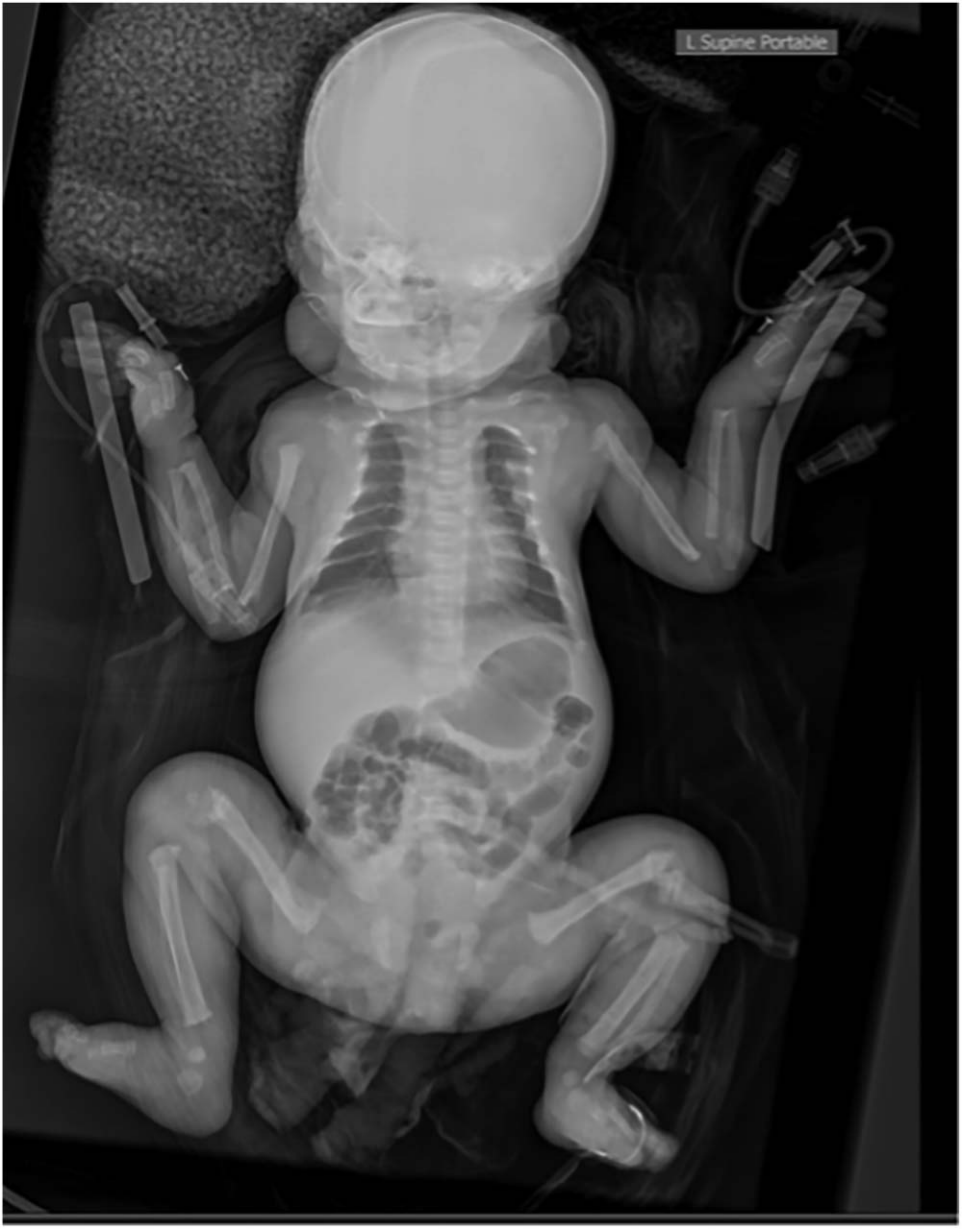
Diffuse osteopenia, multiple healing fractures (ribs, bilateral humeri, bilateral femora and left metatarsals), a bell-shaped thorax, bilateral bowed fibulae, deformed bilateral distal femora and deformed ribs.

Serial serum calcium, phosphate and magnesium levels remained within normal ranges. Ionised calcium was 1.19 mmol/L (reference: 1.15–1.33) on day 0, phosphate was 1.89 mmol/L (1.5–2.6) on day 1, and magnesium was 0.8 mmol/L (0.62–0.91) on day 1. Parathyroid hormone (PTH) was significantly elevated at >263 pmol/L (1.3–6.8) on day 1, with a paired albumin-adjusted calcium of 2.09 mmol/L (1.96–2.66). Total vitamin D was 53 nmol/L (50–220). Alkaline phosphatase (ALP) was initially normal at 276 U/L (145–420) on day 1 but rose to 615 U/L by day 6, peaking at 963 U/L at one month of age (Table 1). Maternal calcium, phosphate, PTH, ALP and vitamin D levels were normal. A postnatal babygram revealed diffuse osteopenia, multiple healing fractures (involving the ribs, bilateral humeri, bilateral femora and left metatarsals), a bell-shaped thorax, bilateral bowed fibulae and deformities of the distal femora and ribs (Fig. 2). These findings were consistent with intrauterine calciopenia leading to metabolic bone disease, rickets with appropriate compensatory PTH elevation.

**Table 1:** Overview of initial investigation results.

Investigation	Timing	Result	Reference range
Ionised calcium, mmol/L	Day 0	1.19	1.15–1.33
Phosphate, mmol/L	Day 1	1.89	1.5–2.6
Magnesium, mmol/L	Day 1	0.8	0.62–0.91
Parathyroid hormone, pmol/L	Day 1	>263	1.3–6.8
Albumin-adjusted calcium, mmol/L	Day 1	2.09	1.96–2.66
Total 25-hydroxyvitamin D, nmol/L	Day 2	53	50–220
Alkaline phosphatase, U/L	Day 1	276	145–420
Alkaline phosphatase, U/L	Day 6	615	145–420
Alkaline phosphatase, U/L	1 month	963	145–420

## Treatment

**Figure 3:**
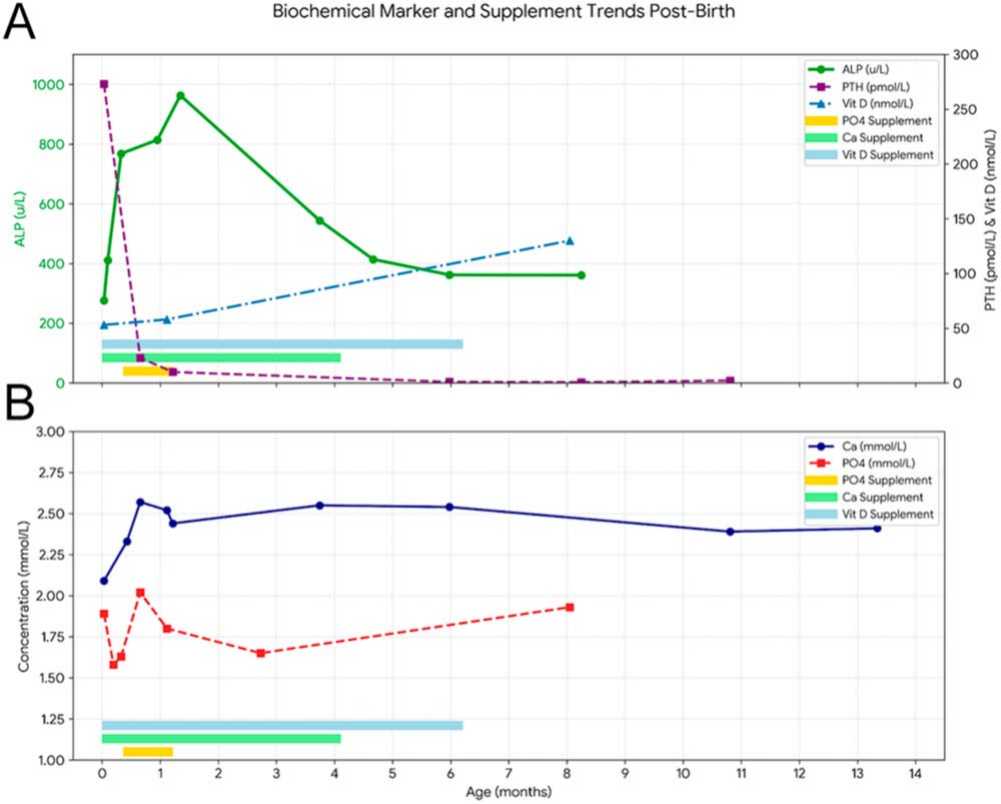
ALP, PTH, vitamin D, Ca and PO4 levels when started supplements over time.

For respiratory distress at birth, she was commenced on non-invasive positive airway pressure support, which was successfully weaned off by two weeks of life. Treatment included oral calcium (50 mg/kg/day), phosphate (0.6 mmol/kg/day) and vitamin D supplementation (400–800 IU/day), all of which were gradually weaned off over the subsequent months (Fig. 3).

## Outcome and follow-up

**Figure 4:**
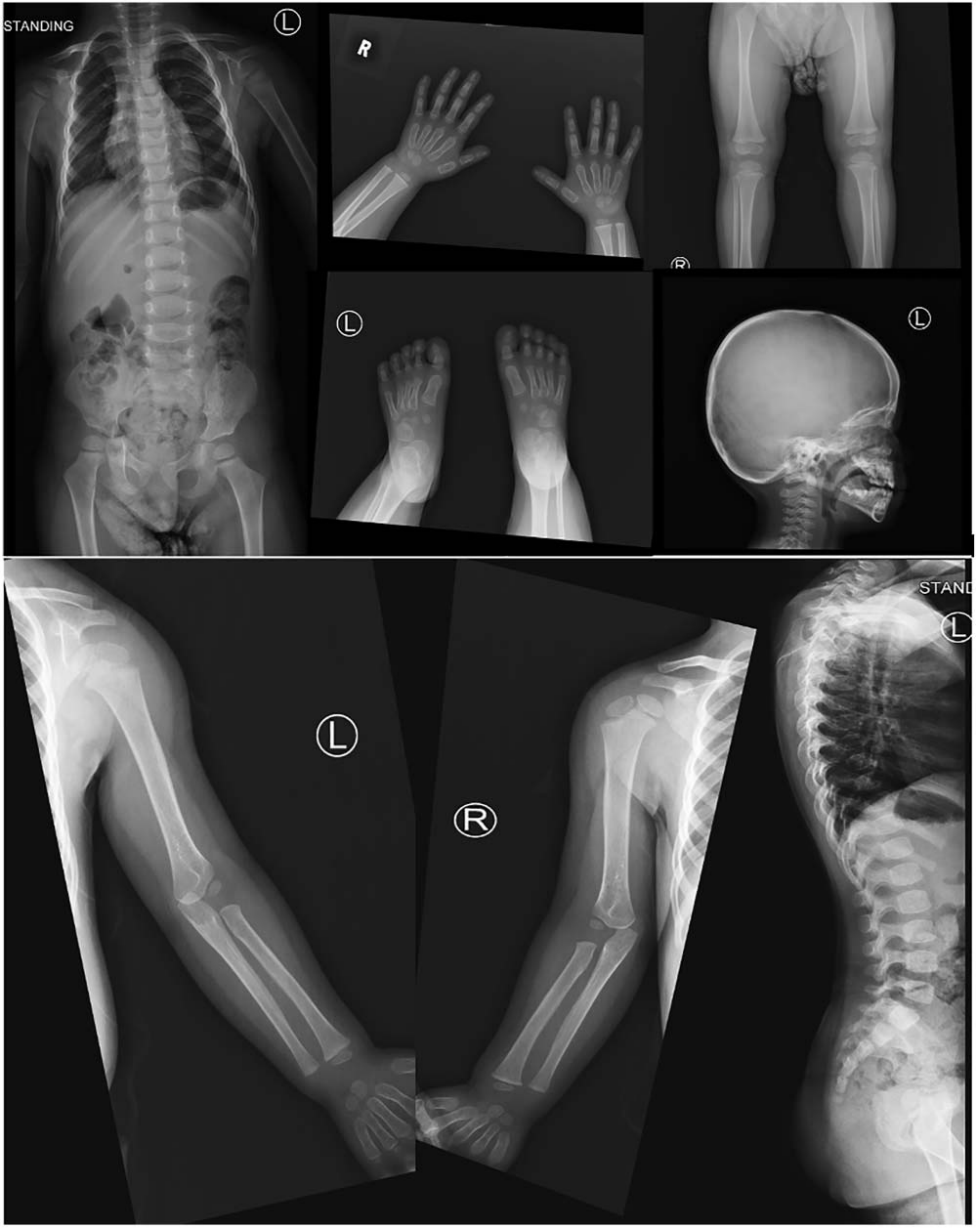
Normal skeletal survey by 2 year old.

The patient was followed in the Endocrine and Neonatal clinics. At 24 months, her weight was 10 kg (25th percentile) and height was 79.2 cm (below 0.4th percentile). Her developmental milestones were age-appropriate; she could run, kick a ball, climb stairs with support, scribble, build towers, use two-word phrases and engage with peers. ALP levels normalised by 4 months of age, and PTH normalised by 2 months. A skeletal survey at 25 months showed resolution of the previous bone deformities (Fig. 4).

## Discussion

We report a Chinese girl with novel TRPV6 variants presenting with short long bones, intrauterine fractures, bone deformities and neonatal secondary hyperparathyroidism. The TRPV6 mutation was first reported by Suzuki *et al.* (1). While this initial report coined the disease ‘Transient Neonatal Hyperparathyroidism’ (OMIM 618188), we recommend renaming this condition according to the dyadic standards of genetic conditions: ‘TRPV6-related intrauterine calciopenic rickets’. This name more appropriately reflects the pathophysiology, as the elevated parathyroid hormone is secondary to the impaired calcium transport, which is the driver of this disorder. To our knowledge, this is the first reported case of intrauterine calciopenic rickets associated with TRPV6 variants in China, and only 13 cases have been described in the existing literature.

During fetal development, skeletal formation and mineralisation require calcium concentrations exceeding basic cellular needs, necessitating energy-dependent placental calcium transport from mother to fetus. This is evidenced by fetal blood calcium levels surpassing maternal levels (2). Postnatally, calcium absorption shifts to passive paracellular intestinal transport (3), explaining why biochemical and skeletal abnormalities in TRPV6 deficiency ameliorate with postnatal calcium, phosphorus and vitamin D supplementation.

The key features of all 14 reported cases are compiled in the Supplementary Table (see section on the Supplementary materials given at the end of the article) (1, 3, 4, 5, 6, 7, 8). The review of the 14 cases shows a relatively balanced gender distribution (eight females and six males), suggesting no sex-linked bias in TRPV6 mutations, although larger cohorts are needed to confirm this. Gestational age ranged from 32 to 40 weeks, with 79% (11/14 cases) exhibiting SGA, due to impaired bone growth secondary to placental calcium transport defect.

19 distinct TRPV6 variants have been identified. Genetically, with one exception (case 14), the disease mostly follows an autosomal recessive inheritance pattern, with compound heterozygous mutations being the most common (71%, 10/14 cases), followed by homozygous mutations (21%, 3/14 cases). The most recurrent variant is p.Gly451Glu (observed in three patients). Variant types include missense (68%, 13/19 cases), frameshift (21%, 4/19 cases), splice-site (5%, 1/19 cases) and nonsense (5%, 1/19 cases) mutations. However, it is difficult to draw any conclusion for genotype–phenotype correlations due to variant heterogeneity.

Prenatally, ultrasound detected abnormalities in 79% (11/14), featuring short long bones, narrow or bell-shaped thorax and polyhydramnios. Postnatally, nearly all infants (92%, 12/13 cases) exhibit respiratory distress secondary to thoracic deformity, requiring transient or prolonged PPV; two cases even required tracheostomy for long-term ventilation. Fractures, predominantly involving ribs and long bones (67%, 8/12 cases), often present prenatally. Skeletal abnormalities usually resolve radiographically between 6 months and 2 years under calcium, phosphate and vitamin D supplementation.

Biochemically, neonates universally present with elevated PTH (14/14 cases), regardless of hypocalcaemia status, which is suggestive of compensatory hyperparathyroidism. ALP was elevated in 86% (12/14 cases), whereas hypocalcaemia, hypophosphatemia and vitamin D deficiency occurred in 64% (9/14), 21% (3/14) and 75% (9/12 of cases), respectively. As shown, this disease entity encompasses a wide spectrum of phenotype, suggesting that disease modifiers, such as maternal calcium status, vitamin D status and population-level calcium intake, may influence the clinical phenotype (5).

Notably**,** 89% **(**8/9 cases) of neonates with vitamin D deficiency exhibited concomitant hypocalcaemia, whereas no hypocalcaemia was observed in those without vitamin D deficiency**.** Prior studies demonstrate that TRPV6 expression is strongly regulated by 1,25-dihydroxyvitamin D (9). A cellular study by Taparia *et al.* found 1,25-dihydroxyvitamin D increases TRPV6 mRNA production in colon cells by 60-fold (10). We hypothesise that hypocalcaemia might arise from the combined effects of TRPV6 dysfunction (a genetic predisposition) and vitamin D deficiency (an environmental factor). This suggests that increased maternal vitamin D supplementation could mitigate phenotypic severity.

Hypocalcaemia typically normalises early with supplementation; however, transient mild hypocalcaemia may persist and generally stabilises by 1 month of age. PTH levels normalise between 1 and 3 months, while ALP levels resolve gradually, typically between 3 and 17 months. Vitamin D levels usually normalise within 3 months. Given these recovery markers are highly sensitive to the dosing and aggressiveness of supplementation, the clinical data should be interpreted with caution.

Diagnosis relies on whole-exome sequencing and prenatal ultrasound findings, reinforcing the need for genetic counselling and multidisciplinary follow-up. Postnatal mineralisation is primarily supported by oral calcium, phosphate and vitamin D supplementation. Based on the case observation between neonatal calcium and vitamin D status, we propose that enhanced maternal vitamin D supplementation during the antenatal period may modulate the severity of TRPV6-related phenotypes and reduce complication risks.

Developmental outcomes are largely favourable: among eight cases with documented follow-up, seven either maintained normal developmental trajectories or achieved age-appropriate milestones by 2 years of age. One case exhibited significant global developmental delay, attributable to a history of cerebral insult, perinatal stroke and a stormy neonatal course (1). Prognosis is generally positive with timely supplementation. No new skeletal abnormalities were reported post-supplementation, underscoring the self-limiting nature of the prenatal metabolic bone disorder.

It is noteworthy that the patient described in this report exhibits a phenotype highly consistent with TRPV6-related intrauterine calciopenic rickets, presenting with all major hallmarks described. These findings align closely with those reported across all other cases in the literature, despite the identified variants currently being classified as VUS. This classification primarily reflects the limited functional and population data available for TRPV6, rather than a lack of clinical correlation. Although the aggregated data reveal consistent phenotypic patterns, several limitations must be acknowledged, include missing data entries – ‘No Report’ in approximately 14% of cases and a potential reporting bias toward severe clinical presentations, which may result in underrepresentation of milder phenotypes. Consequently, cautious interpretation remains warranted. Furthermore, the small cohort size in the existing literature limits the generalisability of these observations and highlights the need for larger, prospective patient registries.

## Supplementary materials



## Declaration of interest

The authors declare that there is no conflict of interest that could be perceived as prejudicing the impartiality of the work reported.

## Funding

This research did not receive any specific grant from any funding agency in the public, commercial or not-for-profit sector.

## Patient consent

Written informed consent for publication of their clinical details was obtained from the patient’s family.

## Author contribution statement

GCY drafted the manuscript, and WSS supervised and revised the manuscript. All authors critically reviewed and endorsed the content of the manuscripts.
