# Supplementary material for: TRPV6-related intrauterine calciopenic rickets: a case report and literature review
Source: Endocrinol Diabetes Metab Case Rep. 2026 Apr 8;2026(2):EDM250192. doi: 10.1530/EDM-25-0192 (PMC13087865; doi:10.1530/EDM-25-0192)
Supplement: Supplementary file 1 [file supplementary_materials.pdf]

Supplementary Table Summary of clinical, biochemical, and genetic features from 14 reported cases of TRPV6-related intrauterine calciopenic rickets, including the present case (Case 1). NR = Not Reported

**Case**	**Gestation, Gender, Body weight (%)**	**Genotype**	**Neonatal Ca^{2+}**	**Neonatal PO4**	**Neonatal ALP (max)**	**Neonatal Vit D**	**Neonatal PTH**	**Ca normalized**	**PO4 normalized**	**ALP normalized**	**PTH normalized**	**Vit D normalized**	**Development**
**1 (PRESENT CASE)**	37+2 weeks Female (<3%)	Compound heterozygous c.1160G>A (p.Cys387Tyr) + c.658C>T (p.Arg220Trp)	Ionised 1.19 mmol/L	1.89 mmol/L	615 IU/L, max 963IU/L at 1m of life (High)	53 nmol/L	>263 pmol/L (High)	/	/	4 months	2 months	/	Normal all along
**2 (ALMIDANI 2020)**	Term Male (<3%)	Homozygous c.593C>G (p. T198R)	0.52 mmol/L (low)	0.4 mmol/L (low)	450 IU/L (High)	NR	7669pmol/L (High)	Day 19	Day 19	NR	Day 28	/	NR
**3 (BURREN 2018)**	Term Female (Normal weight)	Compound heterozygous c.1978G>C (p.Gly660Arg) + c.1528C>T (p.Arg510Ter)	2.43 mmol/L (transiently low at 3-4 week)	1.4 mmol/L (transiently low at 3-4 week)	289 IU/L	29 nmol/L (low)	101 pmol/L (High)	Stable by 4 weeks	Stable by 4 weeks	/	Before 4 months	NR	NR
**4 (YAMASHITA 2019)**	37+0 weeks Female (10%)	Compound heterozygous c.668 T > C (p.Ile223Thr) and c.1282G > A (p.Gly428Arg)	1.92 mmol/L (low)	1.87 mmol/L	998 IU/L (High)	23 nmol/L (low)	2700 pmol/L (High)	2 weeks	/	9 months	6 weeks	6 week	Catch up by 18 months
**5 (SUZUKI 2018-1)**	39+5 weeks Male (10%)	Homozygous c.530_533dup (p.Arg179CysfsTer18)	Ionised 1.27 mmol/L	1.45mmol/L	871 IU/L (High)	99 nmol/L	9.7pmol/L (High)	/	/	Before 3 months	Before 3 months	/	Normal all along
**6 (SUZUKI 2018-2)**	38 week Female (<3%)	Compound heterozygous c.635G > A (p.Cys212Tyr) and c.1282G > A (p.Gly428Arg)	Ionised 1.14mmol/L	1.51mmol/L	576 IU/L (High)	27 nmol/L (low)	238.9 pmol/L (High)	/	/	NR	110 days	NR	Catch up by 2 year old
**7 (SUZUKI 2018-3)**	39+1 week Male (<3%)	Compound heterozygote: c.668 T > C (p.Ile223Thr) and c.1274G > A (p.Arg425Gln)	Ionised 1.2mmol/L	1.2mmol/L	130 IU/L (1 month)	NR	13.8 pmol/L (High)	/	/	/	NR	NR	NR
**8 (SUZUKI 2018-4)**	38 week Female (<3%)	Compound heterozygote: c.1352G > A (p.Gly451Glu) and c.1447C > T (p.Arg483Trp)	Ionised 0.87mmol/L (Low)	1.87mmol/L	1520 IU/L (High)	37.5 nmol/L (low)	154.8 pmol/L (High)	NR	/	NR	2 months	NR	Isolated gross motor delay
**9 (SUZUKI 2018-5)**	34 week Male (87%)	Compound heterozygote: c.1274G > A (p.Arg425Gln) and c.1352G > A	Ionised 0.92mmol/L (Low)	2.23mmol/L	2000 IU/L (High)	20.2 nmol/L (low)	28.7 pmol/L (High)	NR	/	3 months	2 months	3 months	Catch up by 2yr8m

		(p.Gly451Glu)											
**10 (SUZUKI 2018-6)**	38 week Female (<3%)	Compound heterozygous c.607+5G > A and a c.978_979del (p.Asp328*)	2.5mmol/L	1.55mmol/ L	815 IU/L (High)	163 nmol/L	22.8 pmol/L (High)	/	/	NR	NR	/	Significant global developmental delay at 3 year old
**11 (SUZUKI 2020-1)**	36 weeks Male (<3%)	Compound heterozygote: c.854-857del (p.Lys285ArgfsTer9) and c.871G > A (p.Gly291Ser)	Ionised 1.01mmol/L (Low)	1.9 mmol/L	Elevated (no value)	30 nmol/L (Low)	145.4 pmol/L (High)	NR	/	NR	NR	NR	NR
**12 (SUZUKI 2020-2)**	38weeks Male (<3%)	Compound heterozygous: c.1169G > A (p.Arg390His) and c.1352G > A (p.Gly451Glu)	Ionised 0.92mmol/L (Low)	2 mmol/L	Elevated (no value)	34.9 nmol/L (Low)	9.6 pmol/L (High)	Day 1	/	17 months	72 days	NR	NR
**13 (KUMAR 2024)**	Term Female (<3%)	Homozygous: c.1585G>A (p.Asp529Asn)	0.5 mmol/L (low)	1.13 mmol/L (Low)	640 IU/L (High)	9 nmol/L (Low)	302 pmol/L (High)	3 months	3 months	3 months	2 months	3 months	Normal at 3 months
**14 (LAM 2026)**	32 + 6 weeks, Female (Normal weight)	Heterozygous: c.1874T>A (p.Val625Glu) Dominant negative allelevariant	1.98 mmol/L (Low)	1.7 mmol/L	502 U/ (High)	28nmol/L (Low)	230 pmol/L (High)	NR	NR	NR	2 weeks	NR	NR

Supplementary Table (contd.) Summary of clinical, biochemical, and genetic features from 14 reported cases of TRPV6-related intrauterine calciopenic rickets, including the present case (Case 1). NR = Not Reported

Case	Prenatal ultrasound findings	Narrow thorax	Respiratory support duration	Bone fractures	Skeletal resolution age
**1 (PRESENT CASE)**	Short long bones, bell-shaped thorax, polyhydramnios	Yes	Transient CPAP/PPV for 2 weeks then oxygen for 2 weeks	Ribs, humeri, femora, metatarsals	25 months
**2 (ALMIDANI 2020)**	Polyhydramnios, skeletal abnormalities (OI-like)	Yes	Nasal cannula (duration unknown)	Ribs, femur	NR
**3 (BURREN 2018)**	Small chest, short long bones, unusual ribs, polyhydramnios	Yes	Kept ventilated (tracheostomy)	Ribs, metaphyseal, femoral, tibial, and humeral diaphyses	NR
**4 (YAMASHITA 2019)**	Bell-shaped thorax, osteopenia	Yes	"Respiratory support" 2 months	Femur	6 months
**5 (SUZUKI 2018-1)**	Short and bell-shaped chest, short ribs, polyhydramnios	Yes	NR	Ribs, humeri and femoral metaphyses	2 years
**6 (SUZUKI 2018-2)**	Polyhydramnios, short long bones	Yes	Intubated 65 days, CPAP 3 more days	Yes	18 months
**7 (SUZUKI 2018-3)**	Polyhydramnios and short and bowed femora	Yes	CPAP for 10 days	NR	24 months

**8 (SUZUKI 2018-4)**	Polyhydramnios, narrow thorax, rib deformities	Yes	CPAP for 48 days, high flow nasal oxygen supply until 91 days, and nasal oxygen supply until 103 days	NR	Still abnormal by 1 year old. Resolve by 2 year old
**9 (SUZUKI 2018-5)**	Normal	Yes	CPAP with oxygen supplementation for 3 days	Rib fractures	1 year
**10 (SUZUKI 2018-6)**	Normal	Yes	Prolong ventilation, tracheostomy	No	NR
**11 (SUZUKI 2020-1)**	Polyhydramnios and thoracic narrowing with rib deformities.	Yes	Noninvasive positive pressure ventilator was used until the child reached 13 months of age	No	NR
**12 (SUZUKI 2020-2)**	Normal	Yes	CPAP for 6 days	Femoral metaphyseal fracture	21 months
**13 (KUMAR 2024)**	*Polyhydramnios, narrow thorax, short bones, fetal growth restriction*	Yes	CPAP for 10 days	No	Still abnormal at 3 months old
**14 (LAM 2026)**	Narrow and irregular chest wall; normal long bones	Yes	CPAP (duration unknown)	No	NR

References

1. Suzuki Y, Chitayat D, Sawada H, Deardorff MA, McLaughlin HM, Begtrup A, et al. TRPV6 Variants Interfere with Maternal-Fetal Calcium Transport through the Placenta and Cause Transient Neonatal Hyperparathyroidism. The American Journal of Human Genetics. 2018 Jun;102(6):1104–14.

2. Salles JP. Bone metabolism during pregnancy. Annales d'Endocrinologie. 2016 Jun 1;77(2):163–8.

3. Kumar C, Vani S, Deshmukh NN, Omkaram S, Pothala R, Bathina SP, et al. Novel TRPV6 variant linked with transient neonatal hyperparathyroidism. Egypt J Med Hum Genet. 2024 Jun 27;25(1):72.

4. Burren CP, Caswell R, Castle B, Welch CR, Hilliard TN, Smithson SF, et al. TRPV6 compound heterozygous variants result in impaired placental calcium transport and severe undermineralization and dysplasia of the fetal skeleton. American J of Med Genetics Pt A. 2018 Sep;176(9):1950–5.

5. Suzuki Y, Sawada H, Tokumasu T, Suzuki S, Ninomiya S, Shirai M, et al. Novel TRPV6 mutations in the spectrum of transient neonatal hyperparathyroidism. The Journal of Physiological Sciences. 2020;70(1):33.

6. Almidani E, Elsidawi W, Almohamedi A, Bin Ahmed I, Alfadhel A. Case Report of Transient Neonatal Hyperparathyroidism: Medically Free Mother. Cureus [Internet]. 2020 Feb 15 [cited 2025 Aug 24]; Available from: https://www.cureus.com/articles/27865-case-report-of-transient-neonatal-hyperparathyroidism-medically-free-mother

7. Yamashita S, Mizumoto H, Sawada H, Suzuki Y, Hata D. TRPV6 Gene Mutation in a Dizygous Twin With Transient Neonatal Hyperparathyroidism. Journal of the Endocrine Society. 2019 Mar 1;3(3):602–6.

8. Lam Shang Leen J, Suzuki Y, Koh AL, Jayagobi PA, Nishimura G, Lim WK, et al. Transient neonatal hyperparathyroidism caused by a monoallelic TRPV6 dominant negative variant. JBMR Plus. 2026 Jan 9;10(2):ziaf159.

9. Peng JB, Suzuki Y, Gyimesi G, Hediger MA. TRPV5 and TRPV6 Calcium-Selective Channels. In: Kozak JA, Putney JW, editors. Calcium Entry Channels in Non-Excitable Cells [Internet]. Boca Raton (FL): CRC Press/Taylor & Francis; 2018 [cited 2025 Oct 11]. Available from: http://www.ncbi.nlm.nih.gov/books/NBK531440/

10. Taparia S, Fleet JC, Peng JB, Wang XD, Wood RJ. 1,25-Dihydroxyvitamin D and 25-hydroxyvitamin D--mediated regulation of TRPV6 (a putative epithelial calcium channel) mRNA expression in Caco-2 cells. Eur J Nutr. 2006 Jun;45(4):196–204.
